# Searching for a Connection Between the Phenomenon of Transport Exclusion and the Presence of Gastroenterological Symptoms: A Survey of Secondary School Students in Poland

**DOI:** 10.3390/nu18060949

**Published:** 2026-03-17

**Authors:** Hubert Paweł Szyller, Agata Mytych, Gabriela Augustynowicz, Marta Dziedziak, Maria Lasocka, Mikołaj Michałek, Tomasz Pytrus

**Affiliations:** 1 2nd Clinical Department of Paediatrics, Gastroenterology and Nutrition, Wroclaw Medical University, 50-369 Wroclaw, Poland; tomasz.pytrus@umw.edu.pl; 2 Student Scientific Group of Pediatric Gastroenterology and Nutrition, Wroclaw Medical University, 50-369 Wroclaw, Poland; agata.mytych1@gmail.com (A.M.); gabriela.augustynowicz@intena.pl (G.A.); marta.dziedziak@gmail.com (M.D.); marysialasocka@gmail.com (M.L.); demiki40@gmail.com (M.M.)

**Keywords:** gastroenterology, pediatrics, diet, school nutrition, child, abdominal pain, nausea, transportation barriers, dietary pattern

## Abstract

Background: Transport exclusion and difficult access to educational institutions pose a significant problem for maintaining daily routines, sleep patterns and eating habits and are a potential cause of gastroenterological disorders. This study aims to demonstrate the possible associations between transport difficulties and selected gastrointestinal symptoms. Methods: A cross-sectional anonymous online survey was conducted among 650 students aged 15–20 years from urban and rural areas. Data on place of residence, commuting time, wake-up time, breakfast habits, and gastrointestinal symptoms were analyzed by chi-square tests, nonparametric methods, and correlation analyses. Results: The survey demonstrated the association between commutes to school and the occurrence of morning nausea, abdominal pain, and irregular meal patterns. Early wake-up times are also associated with the risk of symptoms. Transport-related factors, particularly long commuting times and early wake-up schedules, are associated with more irregular eating patterns and a higher prevalence of morning gastrointestinal symptoms in adolescents. Conclusions: Transport exclusion may represent an important, yet understudied, factor influencing adolescent health.

## 1. Introduction

Transport poverty, also referred to as transport-related social exclusion, is a concept increasingly used in the scientific literature to describe situations in which limited availability, accessibility, or affordability of transport restricts individuals’ access to essential destinations such as workplaces, healthcare services, administrative institutions, and educational facilities. Among children and adolescents, transport poverty most commonly manifests as spatial or temporal barriers that hinder regular access to educational institutions and other daily activities [1].

This situation is particularly evident in rural areas and in the outskirts of large urban centers, where there is often a lack of sufficiently developed transport infrastructure [2,3,4]. Commuting patterns vary significantly depending on the place of residence. In cities, young people have greater opportunities to use public transport, bicycles, or walk. In rural areas and suburbs, however, commuting by private car and school bus is predominant [2,5,6]. Often, secondary school students have to travel long distances within a given city or to another city to get to school [7]. Such conditions require students to be physically and mentally mobile on a daily basis, as they have to get up very early to get to class on time [8].

Long commutes to school limit young people’s participation in active forms of transport, such as walking or cycling [5]. The share of active transport decreases especially where distances to schools are long [7]. Active transport has a positive impact on students’ physical condition and mental well-being [5]. Students who use active transport are more likely to follow healthy eating habits, while young people who commute passively (by bus or car) are less likely to adopt these habits [6].

Chronic sleep deprivation and stress resulting from limited transport options are significant pathophysiological factors for children’s bodies, contributing to the development of psychosomatic symptoms. Young people are more likely to experience headaches, stomach aches, fatigue, and gastrointestinal disorders [5,9,10]. Sleep problems are very often closely related to poor eating habits, especially skipping breakfast [11]. Skipping breakfast may further exacerbate the above psychosomatic symptoms in adolescents [6,7,12].

In Poland, extensive research on transport exclusion, linked to the analysis of public transport availability and household ownership of personal means of transport, is a new topic that is developing as a result of the significant intensification of the decline in public transport that has been ongoing for over a decade due to the Coronavirus Disease 2019 (COVID-19) pandemic. A report by the Polish Prime Minister’s Office shows a 42% decrease in the total length of bus routes compared to 2014, and also states that over 30% of residents of rural areas and small towns are dissatisfied with public transport services. According to the latest United Nations International Children’s Emergency Fund, od 1953 United Nations Children’s Fund (UNICEF) report, 14% of 12–19 year olds are at risk of transport exclusion, with this figure reaching 26% in peripheral areas. This problem also affects 17% of all secondary school students [1,13].

Although the issue of transport exclusion and long commutes has been widely discussed in the context of access to education and participation in social life, as well as general access to healthcare, the potential health consequences of this phenomenon among adolescents remain largely undescribed, particularly in terms of internal medicine symptoms.

The aim of this study was to examine the association between commuting-related difficulties, wake-up time, and selected gastrointestinal symptoms among secondary school students.

## 2. Materials and Methods

The survey involved students aged 15–20, which in Poland is the age range covered by public high school education. Respondents voluntarily completed an anonymous online survey, preceded by appropriate instructions. In order to maintain diversity in the responses and ensure an appropriate social cross-section, students from schools located in towns and cities of various sizes and locations were invited via schools to participate in the online survey in equal numbers: from the largest cities in the country and their suburbs, through smaller towns that are the main centers of smaller administrative sectors, to small towns and rural areas. The number of inhabitants in the towns where the respondents live and study was taken into account as a differentiating factor in this respect. The sizes of the places of residence proposed in the responses have been adjusted to the administrative divisions and population size of Poland. Each respondent was given the opportunity to contact the authors of the survey, leave comments, ask questions, or request clarification of the questions, which were linguistically adapted to the target group. The surveys were collected in 2021–2022. The inclusion criteria were as follows: being a secondary school student and providing informed consent to participate in the survey. Responses that were incomplete or inconsistent were excluded from the final analysis.

The 36-question form that the respondents answered was divided into three parts:

### 2.1. Basic Information

The questions in this section included age, gender, size of the place of permanent residence, location of the school and its distance from the place of residence, the most frequently chosen means of transport to school, average travel time to school, the most common wake-up time, and the usual start time of school lessons. This section contained 8 questions.

### 2.2. Questions About Morning Eating Habits

This section asked how often the respondent eats breakfast and how often they skip it, as well as the reasons for skipping breakfast. It also asked for their own assessment of the regularity of their breakfast consumption, what drink they choose for breakfast, what foods they eat most often (based on open-ended answers and multiple choices, these were sorted into general groups such as cereal products, dairy products, fruit, and vegetables), whether breakfast is eaten in a comfortable atmosphere without stress resulting from school events (tests, exams, presentations) or transportation to school, and whether they eat a second breakfast, if so, at what time and what it contains and where it comes from (whether it is a ready-made processed product, breakfast prepared at home from freshly purchased ingredients, food bought in stores or eateries, or the school cafeteria). They were also asked what influences their choice of this type of lunch and, given the growing popularity of this practice, whether the respondent counts the calories in their meal and, if so, for what reason (medical, dietary, no specific purpose).

There was also a series of questions related to family eating habits and diets suggested by parents. This section contained 15 questions.

### 2.3. Questions Related to Medical History, with Particular Emphasis on Gastroenterological Complaints

In this part of the survey, respondents were asked to provide information about any chronic diseases and medications they take on a regular basis.

This was followed by a series of questions about any gastroenterological complaints, whether their severity was increased in the morning, and whether the respondent noticed a correlation between the symptoms and the regularity of breakfast consumption. The symptoms included nausea, abdominal pain, vomiting without apparent cause, and stomach discomfort without apparent cause. In the questions, respondents were given the opportunity to provide the frequency and severity of the symptoms mentioned, based on their own assessment, with the degrees of severity explained previously, and whether the symptoms require medication. Respondents were also asked whether they had ever consulted a doctor about these symptoms.

The survey also asked whether the respondent had noticed similar symptoms among their peers. This section contained 13 questions.

### 2.4. Statistical Analysis of the Received Responses

The data were divided into qualitative variables (place of residence, commute time, symptoms) and quantitative variables (age, assessment of meal regularity). The correlations between qualitative variables were analyzed using the χ^2^ test, and in cases of small sample sizes, Fisher’s test was used. To compare the mean values of quantitative variables in multiple groups, analysis of variance (ANOVA) or the Kruskal–Wallis test was used when the parametric assumptions were not met. Correlations between ordinal variables were assessed using Spearman’s coefficient, and the multifactorial influence of predictors on the presence of symptoms was examined using logistic regression. Statistical significance was set at *p* < 0.05. The analyses were performed in Python 3. Artificial intelligence tools were used only for supplementary verification of calculations, while all statistical analyses were conducted using standard statistical procedures.

## 3. Results

### 3.1. Basic and Socio-Demographic Data

As a result of the survey, 659 questionnaires were collected, 9 of which were rejected due to incompleteness. The survey covered a group of 650 students aged 15–20 (M = 16.6 ± 1.3). The majority of respondents were female (74.8%).

Respondents lived mainly in provincial capitals (35.3%) and rural areas (28.5%). The main means of transport used to get to school was public transport (68.0% of all responses). Over 40% of students had a journey time of over 45 min, which indicates significant potential for transport exclusion. Over 45% of respondents wake up before 6:00 a.m. The most common school start times are 7:30 a.m. and 8:00 a.m. (a total of ~72% of respondents). All of the data described above, including the percentage share of possible responses, have been compiled in Table 1, Table 2, Table 3 and Table 4.

### 3.2. Breakfast Dietary Patterns

Breakfast was always eaten (39%) or often (20%), but 25% declared that they rarely or never ate breakfast. The most popular breakfast drinks were water (56%) and tea (27%), with coffee accounting for 11% of choices.

In the multiple-choice question, cereal products (42%) and dairy products (38%) dominated; fruit/vegetables appeared less frequently (12%), and vegetables alone was mentioned by only 7% and ready-to-heat meals by only 1%. After interpreting the open-ended responses and categorizing them, bread/sandwiches (43%) and cereal/porridge (38%) were mentioned most often. A small percentage indicated eggs (4%) or sweet dishes. The similarity of responses in the multiple-choice and open-ended questions confirms the dominance of high-carbohydrate, easily accessible meals. A summary of the multiple-choice results is provided in Table 5. In the case of beverage selection, the responses are summarized in Table 6.

Among the sources of supply for students’ second breakfasts, meals prepared in advance at home are by far the most popular (71.7%). Almost equally popular (approx. 11/12%) is the purchase of breakfast outside of school during the school day in a shop or bar and purchases within the school (canteens, school shops). Ready-made, packaged sweet products have the lowest share in the ranking (5.6%), as summarized in Table 7.

Most respondents (69%; n = 450) declare that they usually eat a second breakfast at school. 201 respondents (31%) skip this meal. The most popular time for eating a second breakfast during the day is between 10:00 and 10:59 a.m.—37.2% of all responses. The time breakdown is shown in Table 8.

### 3.3. Gastroenterological Symptoms

Nausea occurs in approximately 64% of respondents at least occasionally, with 20% complaining of it “often” or “almost always.” Although most students reported “no” experience of morning vomiting, 13% reported recurrent episodes of morning vomiting. In the case of abdominal pain, the distribution was as follows: approximately 20% experience pain “often” or “always”. Among the 545 students who reported any morning symptoms (nausea, stomachaches, or vomiting), only 142 (26.6%) consulted a doctor about these symptoms. These data are presented in Table 9, Table 10, Table 11 and Table 12.

### 3.4. Statistical Correlations Analysis

Seven correlations that fully met the established requirements were considered statistically significant. A result was considered significant only when *p* < 0.05, q < 0.05, the effect size cut-off above was met and sample size rules were satisfied. All other analyses were exploratory.

#### 3.4.1. Place of Residence and Nausea

**Figure 1:**
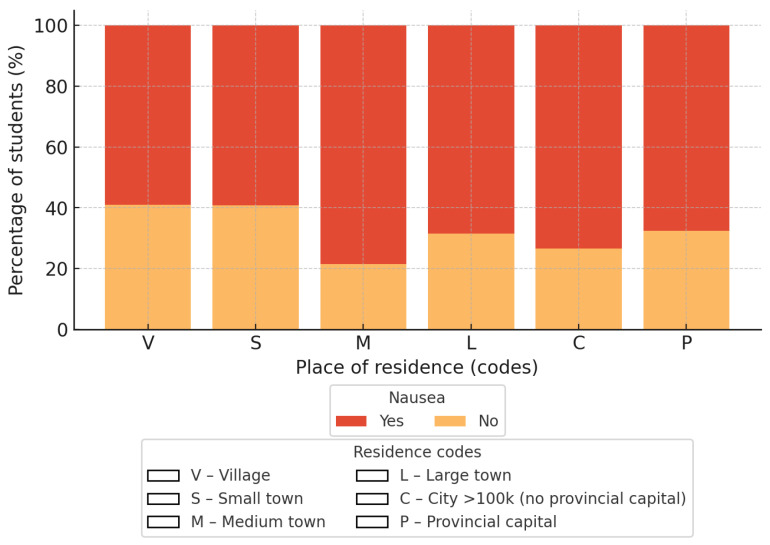
Presentation of the correlation between the size of the place of residence and the presence of any morning nausea. A legend regarding the size of the dwelling place has been included.

The contingency table analysis showed a significant correlation between place of residence and the occurrence of morning sickness (χ^2^ = 11.77; *df* = 5; *p* = 0.038; Cramer V = 0.13). The percentage of students reporting nausea was highest in provincial capitals and lowest in rural areas, indicating variation across residence categories rather than a simple urban–rural gradient on the risk of discomfort after waking up. The results are graphically presented in Figure 1.

In logistic regression analysis (reference category: village), students living in towns with up to 50,000 inhabitants had significantly higher odds of reporting morning nausea (OR 2.58; 95% CI 1.40–4.75). No significant differences were observed for towns with up to 10,000 inhabitants (OR 1.03; 95% CI 0.55–1.90), cities up to 100,000 inhabitants (OR 1.54; 95% CI 0.87–2.73), cities with 100,000–200,000 inhabitants excluding provincial capitals (OR 1.94; 95% CI 0.82–4.59), or provincial capitals with more than 200,000 inhabitants (OR 1.43; 95% CI 0.96–2.14).

#### 3.4.2. Place of Residence and Abdominal Pain

**Figure 2:**
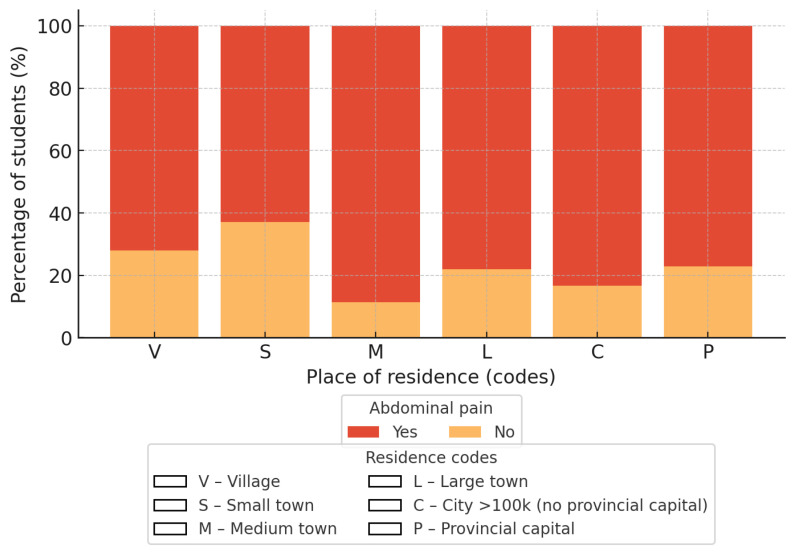
Presentation of the correlation between the size of the place of residence and the presence of any morning abdominal pain. A legend regarding the size of the dwelling place has been included.

A significant association between place of residence and the occurrence of morning abdominal pain was observed (χ^2^ = 14.93; *df* = 5; *p* = 0.011; Cramer V = 0.15). Differences were noted among residence categories, with higher proportions of reported pain in certain urban settings compared with rural areas (Figure 2).

In logistic regression analysis (reference category: rural areas), students living in towns with up to 50,000 inhabitants had significantly higher odds of reporting morning abdominal pain (OR 3.02; 95% CI 1.41–6.48). No significant differences were observed for towns with up to 10,000 inhabitants (OR 0.66; 95% CI 0.35–1.25), cities up to 100,000 inhabitants (OR 1.38; 95% CI 0.73–2.62), cities with 100,000–200,000 inhabitants excluding provincial capitals (OR 1.94; 95% CI 0.71–5.34), or provincial capitals with more than 200,000 inhabitants (OR 1.26; 95% CI 0.81–1.97).

#### 3.4.3. Travel Time and Nausea

**Figure 3:**
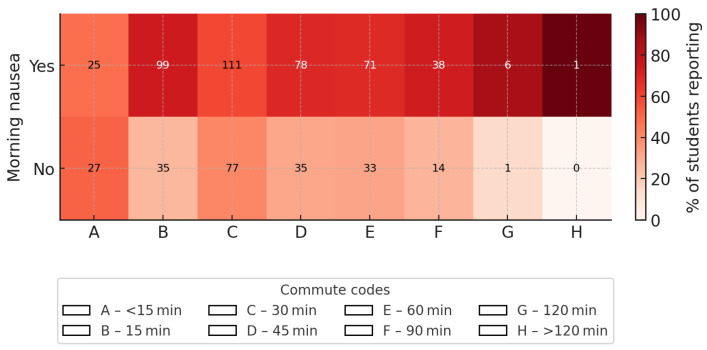
Presentation of the correlation between travel time to school and the occurrence of morning nausea. The size of the group is represented by the colors explained in the legend. The legend also explains the time intervals.

A significant association was observed between travel time to school and the occurrence of morning nausea (χ^2^ = 18.77; *df* = 7; *p* = 0.009; Cramer V = 0.17). Differences were observed across commuting time categories, with generally higher proportions of reported nausea among students with longer travel times (Figure 3).

In logistic regression analysis (reference category: travel time < 15 min), students commuting approximately 45 min (OR 2.41; 95% CI 1.23–4.72), 60 min (OR 2.32; 95% CI 1.17–4.60), and 90 min (OR 2.93; 95% CI 1.29–6.65) had significantly higher odds of reporting morning nausea. A significant elevation was also observed for 10–15 min (OR 3.05; 95% CI 1.57–5.95), whereas the estimate for approximately 30 min was not statistically significant (OR 1.56; 95% CI 0.84–2.88). The odds ratio for approximately 120 min was higher (OR 6.48; 95% CI 0.73–57.66) but imprecise due to the small number of respondents in this category.

Although higher odds were generally observed for longer commuting times, the pattern was not strictly monotonic across all categories, and therefore does not indicate a clear linear dose–response correlation.

#### 3.4.4. Commuting Time and Frequency of Abdominal Pain

**Figure 4:**
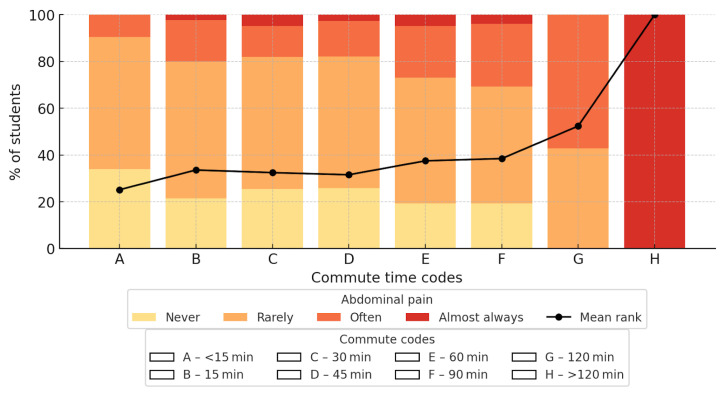
Presentation of the correlation between travel time to school and the occurrence of morning stomach aches. An upward trend is shown as travel time increases. The meaning of colors and codes is explained in the legend.

A significant association was observed between commuting time and the frequency of morning abdominal pain (χ^2^ = 53.26; *df* = 21; *p* < 0.001; Cramer V = 0.165). Differences were observed across commuting time categories, with higher frequencies of reported abdominal pain among students with longer travel times (Figure 4).

In logistic regression analysis (reference category: travel time < 15 min), students commuting approximately 60 min (OR 3.54; 95% CI 1.28–9.79) and 90 min (OR 4.27; 95% CI 1.43–12.73) had significantly higher odds of reporting frequent abdominal pain. A markedly elevated estimate was observed for approximately 120 min (OR 12.80; 95% CI 2.21–74.22), although this result should be interpreted with caution due to the wide confidence interval and small sample size in this category. Shorter commuting times (10–45 min) were associated with elevated but statistically non-significant odds.

Although higher odds were generally observed with increasing commuting time, the pattern was not strictly monotonic across all categories, and estimates for the longest travel groups were affected by limited numbers of respondents.

#### 3.4.5. Commuting Time and Meal Regularity

**Figure 5:**
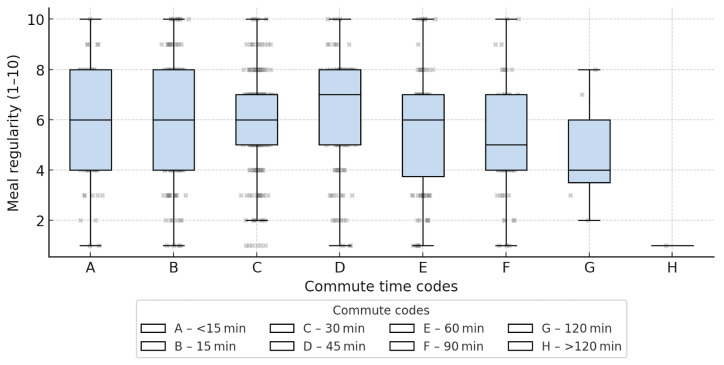
A box plot showing the variation between the regularity of meals consumed and travel time to school.

The Kruskal–Wallis test indicated a statistically significant but small association between commuting time and meal regularity (H = 17.82; *p* = 0.013; η^2^ ≈ 0.017). A weak negative correlation was observed, suggesting slightly lower regularity scores among students with longer commuting times. Differences between groups were modest, and estimates for the longest commuting categories should be interpreted with caution due to small sample sizes (Figure 5).

#### 3.4.6. Wake-Up Time, Nausea and Abdominal Pain

Spearman’s rank correlation analysis demonstrated a weak negative correlation between wake-up time and nausea severity (ρ = −0.08; *p* = 0.046), and a slightly stronger correlation with abdominal pain (ρ = −0.12; *p* = 0.001), indicating more frequent symptoms among students waking earlier.

However, in logistic regression analysis (reference category: 7:00–7:30), wake-up time was not significantly associated with the occurrence of any morning nausea (5:00–5:30: OR 1.49; 95% CI 0.72–3.06; 5:30–6:00: OR 1.19; 95% CI 0.62–2.28; 6:00–6:30: OR 0.95; 95% CI 0.50–1.80; 6:30–7:00: OR 0.97; 95% CI 0.48–1.97; ≥7:30: OR 1.13; 95% CI 0.19–6.76). In contrast, very early wake-up times were associated with significantly higher odds of frequent abdominal pain (often or almost always), particularly for 5:00–5:30 (OR 5.75; 95% CI 1.91–17.30) and 5:30–6:00 (OR 3.20; 95% CI 1.09–9.39), whereas later categories showed elevated but statistically non-significant odds (6:00–6:30: OR 2.51; 95% CI 0.85–7.41; 6:30–7:00: OR 2.33; 95% CI 0.74–7.33; ≥7:30: OR 2.30; 95% CI 0.21–24.80). Overall, the association between earlier wake-up time and gastrointestinal symptoms appears modest for nausea and more pronounced for frequent abdominal pain, particularly in the earliest wake-up categories.

## 4. Discussion

The main purpose of this study was to investigate the associations between difficulties caused by daily commuting to an educational institution and selected gastroenterological symptoms occurring in the pediatric population. To the best of our knowledge, this is one of the few studies directly comparing so-called transport exclusion with the presence or severity of gastrointestinal symptoms. Statistically significant associations between commuting-related factors and self-reported gastrointestinal symptoms were observed.

The results indicate a significant association between commuting time to school and the occurrence of somatic symptoms. A clear correlation was observed (χ^2^ = 18.13; *df* = 7; *p* = 0.011; Cramer V = 0.17) between commuting duration and nausea. Students whose journey lasted at least 45 min reported nausea much more frequently compared to their peers commuting less than 15 min, suggesting that longer commuting times may be associated with higher symptom frequency. A similar correlation was noted in the case of abdominal pain: the frequency of its occurrence increased with commuting time (χ^2^ = 53.26; *df* = 21; *p* = 0.00013; Cramer V = 0.17). Particularly marked differences were found among students commuting an hour or more, who most often reported their symptoms as occurring “often” or “almost always.”

Research on the association between commuting time and somatic complaints in children and adolescents remains limited. Valuable insights are provided by a case study conducted in Dhaka by Rahman et al. [14], which showed that stress levels in children increased with longer commuting times to school. This was accompanied by both somatic symptoms (including headaches and abdominal pain) and emotional and behavioral difficulties such as irritability, anxiety, concentration problems, and sleep disturbances. These findings suggest that long commutes may not only exacerbate physical complaints but also negatively affect students’ psychological functioning [14].

The analysis also revealed a correlation between commuting time and meal regularity. The Kruskal–Wallis test confirmed that meal regularity (scale 1–10) systematically decreased with longer travel times (H = 17.82; *p* = 0.013; η^2^ ≈ 0.017). The median score dropped from 8.0 in the group commuting less than 15 min to 6.5 among those traveling at least one hour. This finding suggests that long commuting times may contribute to meal pattern disruptions and less favorable dietary habits.

Our results are consistent with observations from international studies, although available data remain limited. In Norway, 22% of high school students regularly skipped breakfast, with 59% connecting it with lack of time, mainly due to prioritizing extra sleep or long commutes [15]. Similarly, in a U.S. study, women with commutes of ≥90 min were 20 percentage points more likely to skip breakfast compared with those with shorter travel times [16].

Wake-up time was also associated with the frequency of nausea and abdominal pain. Spearman’s rank correlation showed a weak but significant negative association with nausea (ρ = –0.08; *p* = 0.046), indicating that earlier waking was linked to more frequent symptoms. A stronger correlation was observed for abdominal pain (ρ = –0.12; *p* = 0.001), with students waking before 5:30 reporting, on average, pain scores 0.4 points higher (on a 0–3 scale) compared with those waking after 6:30.

Yeo et al. found that shorter sleep duration on school nights was associated with poorer self-rated health, an increased risk of being overweight (ORadj = 2.56; 95% CI 1.39–4.70), and a higher likelihood of depressive symptoms. Among the factors contributing to poor sleep, the authors listed later preferred bedtime, longer study hours, earlier school start times, and longer commuting times, all of which may hinder maintaining adequate sleep [17]. Similarly, Smaldone et al. reported that insufficient sleep increased the risk of atopic conditions and frequent, severe headaches [18], while Schlieber and Han emphasized the negative impact of short and irregular sleep on children’s physical health, cognitive functioning, and socioemotional processes [19]. Furthermore, Gale et al. demonstrated that irregular sleep was associated with unhealthy food choices, reduced physical activity, increased ghrelin and cortisol levels, and weight gain in adolescents [20].

Closely related to our findings are observations regarding the association between meal regularity and child health. Studies consistently indicate that skipping meals, particularly breakfast and lunch, has a significant impact on the physical and mental health and overall functioning of children and adolescents [21,22,23,24,25,26,27,28,29]. Reported associations include reduced insulin sensitivity and metabolic disturbances [21] as well as a higher prevalence of gastroesophageal reflux [26]. Links have also been noted with abdominal and back pain, sleep difficulties, nervousness, irritability, and headaches [24,27]. Other studies point to associations with reduced self-esteem [22], increased depressive symptoms, stress, and suicidal ideation, as well as a greater tendency toward risky behaviors, including alcohol and drug use and a sedentary lifestyle [23].

Another noteworthy and somewhat unexpected observation from our study is that children residing in large cities report morning nausea more frequently than their peers living in rural areas (χ^2^ = 11.77; *df* = 5; *p* = 0.038; Cramer V = 0.13). The results indicate that the proportion of children experiencing morning nausea is approximately 20% higher among students from medium-sized cities than among those from rural areas.

A potential explanation for this correlation may be the greater availability of processed foods in urban settings compared with rural ones. Studies have shown that the share of ultra-processed foods (UPFs) in the diet is higher in urban environments, likely due to more intensive marketing and greater availability of food outlets offering such products [30]. Diets rich in ultra-processed foods are characterized by low fiber content and high amounts of additives, fats, sugars, and emulsifiers. These substances alter the gut microbiota composition—reducing the abundance of beneficial probiotic bacteria (e.g., Bifidobacterium, Lactobacillus) while increasing pro-inflammatory taxa (e.g., Proteobacteria). As a result, there is overproduction of gases and bacterial toxins, fermentation disturbances, and delayed gastric emptying [31,32], which in turn may lead to sensations of fullness, nausea, and abdominal discomfort [33].

Another possible explanation involves greater air pollution and smog exposure in urban areas compared with rural regions. Evidence indicates that higher rates of gastro-intestinal disorders among children correlate with increased concentrations of air pollu-tants (PM_2.5_, NO_2_, ozone) typical of large cities [34,35]. Analyses of global data on PM_2.5_ exposure—the most harmful airborne pollutant—demonstrate a substantial urban–rural disparity [36]. This discrepancy is largely driven by heavier traffic and concentrated industrial emissions in cities, which generate higher local concentrations of fine particulate matter than most rural areas [37].

Several mechanisms have been proposed to explain how air pollution contributes to dyspeptic symptoms in children, including alterations in gut microbiota composition, in-creased intestinal permeability, and low-grade intestinal inflammation [38]. Additionally, long-term exposure to polluted air has been associated with an increased risk of irritable bowel syndrome (IBS) in children, a condition that can also manifest with nausea [39].

Finally, morning nausea may be linked to stressful early-day routines associated with the fast-paced urban lifestyle. There is evidence that psychosocial stressors and emotional tension—more prevalent in urban environments—are correlated with functional gastrointestinal disorders and nausea in children, which may represent a third mechanism explaining the observed differences between urban and rural populations [40,41].

### 4.1. Possible Pathophysiological Explanations

In the pathogenesis of nausea and abdominal pain in children, two integrated neuroendocrine systems play a key role, the gut–brain axis (GBA) and the hypothalamic–pituitary–adrenal (HPA) axis, which jointly mediate the interactions between the nervous, hormonal, and immune systems [42,43]. The HPA axis plays a fundamental role in regulating the stress response, and its activation leads to cortisol secretion, which in the short term facilitates adaptation to stress; however, under chronic stimulation it induces neuroendocrine dysregulation and increased visceral reactivity [44,45]. Gulewitsch et al. demonstrated that children with functional abdominal pain or irritable bowel syndrome exhibit reduced HPA axis reactivity to stress, suggesting exhaustion of compensatory mechanisms and disturbances in cortisol feedback regulation—a phenomenon consistent with prolonged HPA activation and stress-induced dysregulation [46].

At the same time, chronic stress may lead to cortisol hypersecretion and increased sympathetic activity, which disrupt intestinal motility, elevate smooth muscle tone, and cause symptoms such as nausea, abdominal pain, or discomfort [47]. Under conditions of prolonged stress, the circadian rhythmicity of the HPA axis is lost, and sleep disturbances exacerbate hormonal instability. Pesonen et al. observed that children with shorter sleep duration and lower sleep efficiency exhibit increased HPA axis activity, reflected by higher cortisol levels upon awakening and throughout the day [48]. Such dysregulation enhances the perception of visceral pain and contributes to disturbances in appetite and bowel habits, particularly in the context of chronic sleep deprivation and early waking hours [49].

The second key mechanism is dysfunction of the gut–brain axis (GBA), which connects the gut and the central nervous system via neuronal, hormonal, immunological, and microbiotic pathways [42,43,50]. The term “gut–brain axis” dates back to the 19th century and originated from observations that stressful situations were often associated with gastrointestinal symptoms [47]. Kaczmarczyk et al. demonstrated that the composition of the gut microbiota in early childhood correlates with intestinal barrier permeability (measured by zonulin levels) and fecal calprotectin, highlighting the role of the microbiome in shaping inflammatory responses and maintaining intestinal epithelial integrity [50]. Microbiota disturbances—resulting from diet, stress, or irregular meal patterns—lead to increased mucosal permeability and antigen translocation, which enhances afferent pain signaling via the vagus nerve to the central nervous system [51]. In the context of chronic stress, epigenetic dysregulation in intestinal epithelial cells has also been observed, along with downregulation of tight junction proteins and increased intestinal permeability, promoting visceral hyperalgesia (enhanced abdominal pain) and perpetuating GBA dysfunction [47]. This aspect is described in more detail below.

Martin and Mayer noted that the intestinal microbiota also regulates the expression of neurotransmitters such as serotonin and γ-aminobutyric acid (GABA), which mediate control of gastrointestinal motility and visceral pain perception [43]. Dysbiosis and the associated inflammatory state may alter serotonergic receptor sensitivity and activate microglia, promoting both peripheral and central sensitization [45,52]. Under chronic stress, activation of the HPA axis further impairs intestinal barrier function via glucocorticoid and pro-inflammatory cytokine activity, creating a vicious cycle of bidirectional interactions between the brain and the gut [45].

These mechanisms are particularly pronounced in children and adolescents exposed to environmental factors such as long school commutes, irregular meals, and reduced sleep duration, which promote HPA axis activation and destabilization of circadian rhythms [48,49]. As a consequence, persistent neuroendocrine and neuroimmune dysregulation develops, perpetuating symptoms of nausea, abdominal pain, and appetite disturbances [53].

### 4.2. Circadian Disruption, Stress-Related Gut–Brain Axis Activation, and Autonomic Dysregulation as Potential Physiological Pathways Linking Extended Commuting and Early Wake Times to Gastrointestinal Dysfunction

Circadian rhythms are endogenous, approximately 24 h oscillations in physiology and behavior regulated by molecular clocks expressed in nearly all mammalian cells, including those of the gastrointestinal (GI) tract [54,55,56]. These rhythms are coordinated centrally by the suprachiasmatic nucleus of the hypothalamus and synchronized with peripheral clocks in GI tissues, orchestrating digestive functions in alignment with environmental cues such as light exposure, sleep–wake cycles, and feeding times [54,55,56]. GI physiology exhibits daily fluctuations arising from multiple interacting mechanisms, including intrinsic circadian regulation (e.g., rhythmic expression of transporters and clock-controlled genes), downstream physiological effects of circadian signaling such as rhythmic hormone secretion and core body temperature oscillations, behavioral cycles including rest–activity patterns, and responses to external cues [54,55,56,57,58]. A substantial proportion of GI functions demonstrate predictable diurnal variation, including epithelial cell proliferation, motility, hormone secretion, gastric acid production, nutrient absorption, intestinal permeability, microbiome composition and activity, and mucosal immune responses [54,55,56,57,58].

Circadian desynchronization is associated with functional gastrointestinal disturbances, such as abdominal pain, constipation, and diarrhea, and has also been implicated in metabolic disorders including obesity and nonalcoholic fatty liver disease [54,55,56,57,58]. Meal timing represents a particularly important regulator of circadian biology, influencing sleep–wake rhythms, thermoregulation, and metabolic processes. The concept of chrono-nutrition emphasizes alignment of food intake with endogenous rhythms, considering not only dietary composition and quantity but also timing. Individuals with a later chronotype often exhibit a higher body mass index, likely due to greater caloric consumption later in the day, highlighting the importance of temporal eating patterns in obesity and metabolic disease [54,55,56,57,58].

Chronic psychosocial stress resulting from long commutes and early wake-up times is a potential activator of the gut–brain axis (GBA) through modulation of the hypothalamic–pituitary–adrenal (HPA) axis and the autonomic nervous system [59,60]. HPA activation leads to increased secretion of cortisol and catecholamines (norepinephrine, epinephrine), which modulate intestinal functions, including motility, intestinal epithelial permeability, and immune response. As a result, the stomach empties more slowly, intestinal transit is accelerated, and intestinal epithelial permeability increases [60,61,62].

Chronic stress in experimental mouse models increased intestinal permeability (leaky gut) by downregulating tight junction proteins [61,63,64,65,66]. At the same time, stress induces neuroimmunological modulation of the intestine, including mast cell activation and the release of neuropeptides such as substance P, Calcitonin Gene-Related Peptide (CGRP), leading to mast cell degranulation and increased secretion of histamine and proteases (tryptase, chymase), which activate PAR-2 receptors on sensory neurons and adjacent mast cells [67,68,69]. Such interactions create a neuroinflammatory loop that perpetuates inflammation and visceral hypersensitivity, lowering the pain threshold even with mild stimuli [70].

The gut microbiota is another key element of the gut–brain axis modulated by stress. Chronic stress alters the composition and diversity of the microflora, reducing the number of beneficial bacteria (Lactobacillus, Bifidobacterium) and favoring pathogens (Proteobacteria) [71,72,73]. Such disturbances lead to a decrease in the production of short-chain fatty acids (SCFAs), which are crucial for the integrity of the intestinal barrier and the modulation of inflammation [71,72,73]. Chronic stress may also affect the Firmicutes/Bacteroidetes ratio in adolescents [74]. Intestinal microbiota dysbiosis can modify intestinal metabolism, including the production of organic acids, amino acids, and neuroactive metabolites, which affects the gut–brain axis, linking intestinal and neurological functions and modulating intestinal motility, appetite, nervous system functions, and immune response [73].

In adolescents, HPA axis activation under stress is particularly intense due to increased hormonal reactivity during this developmental period [75]. Sleep deprivation, resulting, for example, from early rising, further intensifies the HPA response to psychosocial stress [76].

Stress may also influence eating behavior, as individuals experiencing persistent stress frequently report increased appetite and cravings for energy-dense “comfort foods” high in sugar, fat, and salt. These dietary patterns are associated with weight gain and metabolic disturbances and are partly mediated by stress-related hormonal responses involving insulin, cortisol, and ghrelin. Although the consumption of highly palatable foods may provide short-term relief from stress, evidence suggests that such foods can enhance autonomic activation, stimulate HPA axis activity, and reinforce maladaptive eating behaviors. Together, circadian misalignment, stress-induced gut–brain axis activation, autonomic dysregulation, and altered eating patterns may act synergistically to disrupt gastrointestinal homeostasis, providing a plausible physiological pathway linking extended commuting and early wake times to gastrointestinal dysfunction [59,77,78].

### 4.3. Supplementary Observations on Dietary Habits

A positively surprising finding emerging from our study appears to be that Polish students demonstrate generally healthy eating habits. Among the meals consumed, wheat-based products (69.4%) and dairy products (64.5%) predominated, while water was the most frequently chosen beverage for breakfast (56.7%). It has been shown that the consumption of cereal-based breakfast foods, including their whole-grain variants, is associated with a more favorable BMI and lower prevalence of overweight among children and adolescents in observational studies [79]. In addition, the intake of cereal products, which are rich in B-group vitamins and soluble fiber, reduces LDL cholesterol and plasma homocysteine levels, thereby contributing to a decreased risk of hypertension and type 2 diabetes in this population [80].

Furthermore, studies conducted among school-aged children have demonstrated that regular consumption of breakfast rich in whole-grain wheat products supports cognitive function and academic achievement, and is associated with greater life satisfaction [81,82]. Drinking water with breakfast also represents a beneficial health behavior, which can be noted as another positive pattern among Polish students. It has been demonstrated that adequate hydration in the morning supports cognitive performance, particularly short-term memory, in children [83,84].

Convenience foods, typically classified as highly processed products, were chosen by only 4.4% of surveyed students, further confirming that Polish pupils display a relatively healthy dietary pattern. For the mid-morning meal, as many as 71.7% of respondents reported bringing home-prepared meals. Ready-to-heat meals often contain high amounts of salt, saturated fats, and simple sugars, while being poor in vegetable content and of generally lower nutritional quality. Consequently, such food consumption contributes to an increased risk of hypertension, dyslipidemia, and weight gain [33,85].

Moreover, the higher energy density and lower nutritional quality of convenience foods result in reduced satiety and increased portion sizes, leading to excessive energy intake and, consequently, overweight or obesity—conditions increasingly observed among children [86,87,88].

In contrast, preparing meals at home allows for better control over the amounts of salt, fat, and sugar used in cooking. Home-cooked dishes are also more frequently enriched with vegetables and fruits [63]. It has been demonstrated that home-based diets, associated with higher meal quality, are linked to a lower prevalence of obesity [89].

### 4.4. Strengths and Weaknesses of the Study

The credibility of this study is enhanced by the significant research group size of over 600 respondents. The diversity of the respondents’ places of residence is also important, as it was possible to reach residents of both highly urbanized centers and those located far from larger urban centers, such as villages.

The present study is not without its limitations.

The first and most obvious limitation is the skewed gender structure. There is a clear predominance of girls among the respondents (74.8%), which makes the results less reliable for the general population. There may also be bias due to the imperfections of the participant selection process, despite efforts to include the widest possible range of diverse recipients.

Another significant limitation is the unvalidated survey questionnaire. A set of questions based on the Rome IV functional disorder guidelines was used. Before conducting the study, a pilot study was carried out on clinic patients to determine the clarity and comprehensibility of the questions asked. It should be noted that the reported symptoms were based on the patients’ own perception of their symptoms, which reduces the value of the clinical assessment.

Another important limitation is the lack of a multidimensional analysis of additional factors contributing to communication exclusion, such as socio-economic factors and comorbidities or the coexistence of symptoms with stressful events. This limits the interpretability and independence of the presented results. The study did not include multivariate analyses adjusting for potential confounding factors such as socio-economic status, diet, stress levels, sleep quality, or comorbidities. Therefore, these factors may have influenced the observed associations. In a study constructed in this way, only associations can be identified, excluding causal relationships. The results should therefore be interpreted as a basis for further analysis of the problem described.

### 4.5. Solutions Proposed in the Literature

Solutions limiting the negative effects of long commutes to school must work on many levels. They must combine organizational changes at the system level with environmental interventions and social support. Delaying the start of lessons improves the length and quality of pupils’ sleep. It also contributes to their improved mood and significantly reduces the somatic and psychological symptoms associated with early rising [90]. In addition, sleep hygiene education programs in schools show that having this knowledge significantly improves both the length of sleep and sleep-related habits [91,92]. Where commuting makes it difficult to eat a regular morning meal at home, interventions to increase participation in school breakfasts (e.g., morning canteens, grab-n-go breakfasts) result in a reduction in calorie deficit and attention deficit resulting from feelings of hunger [93]. The effectiveness of promoting active transport (walking/cycling) in places where distances and conditions allow it, through school programs and local social campaigns, is not fully understood and requires further research. At present, it depends mainly on simultaneous investments in infrastructure (pavements, cycle paths, safe road crossings) [94].

At the local policy level, the introduction and funding of Safe Routes to School initiatives and related transport regulations improves road safety on the way to school—especially if the policy is coordinated with spatial planning and school transport schedules [95]. Finally, interventions must take into account parental and social barriers such as safety concerns and access to childcare. Information campaigns, local consultations and solutions such as walking school buses and bicycle patrols facilitate the implementation of changes and increase community acceptance [96,97].

## 5. Conclusions

The main aim of this study was to attempt to demonstrate a link between difficult commutes to school for teenagers and the presence and severity of selected gastroentero logical symptoms. It was demonstrated that longer commutes increase the risk of more frequent nausea, morning stomach aches and disruption of eating habits, which indicates an epidemiological need for further research in this area to deepen knowledge about the phenomenon described and develop possible solutions. The next stages of research should be to analyze the results of metabolic stress markers, which may provide information on the body’s biochemical response to the prolonged psychophysical stress to which a child’s body is subjected in such conditions, and to conduct extensive research on factors influencing transport exclusion, socioeconomic factors, health status, location, population distribution and infrastructure.

## Figures and Tables

**Table 1:** Place of residence.

Category	%
Provincial capital (>200k)	35.3
Village (<5k)	28.5
Medium town (≤50k)	12.1
Large town (≤100k)	11.2
Small town (≤10k)	8.3
City 100–200k (non-provincial)	4.6

**Table 2:** Mode of transport to school.

Category	%
Public transport (bus/tram/metro)	68.0
Car (driver/passenger)	16.1
On foot/bicycle	15.8

**Table 3:** Commute time category.

Category	%
<15 min	8.1
≈15 min	20.6
≈30 min	28.9
≈45 min	17.3
≈60 min	15.9
≈90 min	8.0
≈120 min	1.1
>120 min	0.2

**Table 4:** Wake-up interval.

Category	%
5:00–5:30 a.m.	15.6
5:30–6:00 a.m.	29.6
6:00–6:30 a.m.	30.8
6:30–7:00 a.m.	15.5
7:00–7:30 a.m.	7.7
7:30–8:00 a.m.	0.8
Later than 8:00 a.m.	0.2

**Table 5:** Categories of most frequently chosen types of breakfast meal (multiple choices).

Category/Response	%
Cereals/Bread products	42
Dairy (milk, yogurt, cheese, eggs)	38
Fruits/fruit purées	12
Vegetables alone	7
Convenience, ready-to-heat foods	1

**Table 6:** A summary of the types of drinks chosen for breakfast.

Category/Response	%
Water	56.7
Tea	27.1
Coffee (incl. grain coffee)	11.0
Milk (plain/with cereal)	2.9
Juice	1.2
Energy drink	0.6
Soda/fizzy drinks	0.5
Nothing	0.0
Other/mixed answers	0.0

**Table 7:** List of sources of supply for school second breakfast.

Category/Response	%
Prepared at home (sandwiches, etc.)	71.7
Bought at school (canteen/shop/vending)	10.9
Bought outside school (store/bar)	11.8
Pre-packed sweets brought from home	5.6

**Table 8:** Second breakfast timing summary.

Category/Response	%
07:00–07:59	0.5
08:00–08:59	0.5
09:00–09:59	9.7
10:00–10:59	37.2
11:00–11:59	26.3
12:00–12:59	21.5
13:00–13:59	3.9
≥14:00	0.5

**Table 9:** Frequency of morning nausea.

Response Category	%
Never	34.0
Rarely	44.6
Sometimes	0.2
Often	14.8
Almost always	6.6

**Table 10:** Responses to the question about the presence of recurrent episodes of morning sickness.

Response Category	%
Yes	13.0
No	87.0

**Table 11:** Morning abdominal pain frequency.

Response Category	%
Never	23.6
Rarely	55.7
Often	17.2
Almost always	3.5

**Table 12:** Responses from respondents reporting whether they consulted with a doctor.

Response Category	%
Yes	26.6
No	73.4

## Data Availability

The data presented in this study are available upon request from the corresponding author. The data are not publicly available due to restrictions of privacy and ethics that apply to the availability of these data.
